# A New Disease for Europe of *Ficus microcarpa* Caused by *Botryosphaeriaceae* Species

**DOI:** 10.3390/plants11060727

**Published:** 2022-03-09

**Authors:** Alberto Fiorenza, Dalia Aiello, Mariangela Benedetta Costanzo, Giorgio Gusella, Giancarlo Polizzi

**Affiliations:** Department of Agriculture, Food and Environment (Di3A), University of Catania, Via S. Sofia 100, 95123 Catania, Italy; alberto.fiorenza@phd.unict.it (A.F.); dalia.aiello@unict.it (D.A.); benedettacst@gmail.com (M.B.C.); gpolizzi@unict.it (G.P.)

**Keywords:** canker, dieback, Indian laurel-leaf fig, *Ficus microcarpa*, *Botryosphaeriaceae*, phylogeny

## Abstract

The Indian laurel-leaf fig (*Ficus microcarpa*) is an important ornamental tree widely distributed in the urban areas of Italy. Surveys conducted in 2019 and 2020 on several tree-lined streets, squares, and public parks in Catania and Siracusa provinces (Sicily, southern Italy) revealed the presence of a new disease on mature trees. About 9% of approximately 450 mature plants showed extensive branch cankers and dieback. Isolations from woody tissues obtained from ten symptomatic plants consistently yielded species belonging to the *Botryosphaeriaceae* family. The identification of the recovered fungal isolates was based on a multi-loci phylogenetic (maximum parsimony and maximum likelihood) approach of the ITS, *tef1*-α, and *tub2* gene regions. The results of the analyses confirmed the presence of three species: *Botryosphaeria dothidea*, *Neofusicoccum mediterraneum*, and *N. parvum*. Pathogenicity tests were conducted on potted, healthy, 4-year-old trees using the mycelial plug technique. The inoculation experiments revealed that all the *Botryosphaeriaceae* species identified in this study were pathogenic to this host. Previous studies conducted in California showed similar disease caused by *Botryosphaeriaceae* spp., and the pathogenic role of these fungi was demonstrated. To our knowledge, this is the first report of *Botryosphaeriaceae* affecting *Ficus microcarpa* in Europe.

## 1. Introduction

*Ficus microcarpa*, commonly known as Chinese or Malayan banyan, Indian laurel-leaf fig, and curtain fig, is a widely distributed evergreen ornamental species belonging to the family *Moraceae*, native to Ceylon, India, southern China, the Ryukyu Islands, Australia, and New Caledonia [1]. It is considered one of the most common urban trees in warm climates worldwide [2]. Moreover, *F. microcarpa* is also well known as an invader species due to its ability to grow in inhospitable places, its large fruit production, and its numerous dispersal agents (birds, bats, rodents, and others) [3]. Many *Ficus* spp. were introduced in southern Italy as ornamental species; they are now common in many urban areas and viewed as an important form of historical heritage [4]. Parks and gardens in urban areas are of significant value for all people living their daily lives in the cities. Urban trees have a positive impact on reducing heat, providing a convenient shelter, reducing wind velocity, and increasing the aesthetic value of the landscape [5,6,7]. In addition, most people living in cities deal with schedules, work, appointments, meetings, etc., and urban parks and open spaces positively affect mental health [8]. Thus, it is important not to underestimate the health of urban trees.

According to Fungal Database 53 records of fungus association with this host have been reported worldwide [9]. Among these, particular attention is given to species belonging to *Botryosphaeriaceae*. In fact, diseases caused by *Botryosphaeriaceae* are drawing the attention of the researchers worldwide, since they are a significant threat to many crops, especially in Mediterranean climates [10]. *Botryosphaeriaceae* include a large group of diverse fungal species, distributed all over the world. These fungi are well known as plant pathogens, endophytes, and saprophytes of woody hosts [11]. Due to their role as plant pathogens, these fungal species have been studied for a long time, and their impact on forestry and agricultural production is well known [12]. *Botryosphaeriaceae* induce severe symptoms, such as branch, shoot, and trunk cankers, and blight fruits and leaves.

*Botryosphaeriaceae* disease studies on *Ficus* spp., including the cultivated common fig (*F. carica*), have been published worldwide, showing that *Botryosphaeriaceae* and *Diaporthaceae* spp. are involved in complex diseases [13,14,15,16,17,18,19,20,21,22,23,24,25,26,27,28,29]. *Botryosphaeriaceae* cause polyetic epidemics (2–3 cycles per season); thus, the progress of epidemics may extend for several years [10]. In addition, *Botryosphaeriaceae*, characterized by a wide host range, can easily jump from one host to another; this is particularly evident in Mediterranean landscapes, where different crops are cultivated nearby [10]. Especially in the case of urban environments, it must be remembered that dangerous situations are related to the health status of the trees. Therefore, it is important to monitor the health of trees before they become hazardous [30]. Surveys conducted in the metropolitan area of Catania and Siracusa (Sicily), during 2019 and 2020 revealed many *F. microcarpa* distributed among numerous metropolitan areas, including gardens, public parks, tree-lined streets, and squares, showing severe symptoms of branch cankers and dieback. The aims of this study were to (i) investigate the etiology of the disease by (ii) characterizing the fungal isolates recovered from diseased trees based on a multi-loci phylogenetic analysis and (iii) assess their pathogenicity.

## 2. Results

### 2.1. Surveys and Fungal Isolations

**Figure 1:**
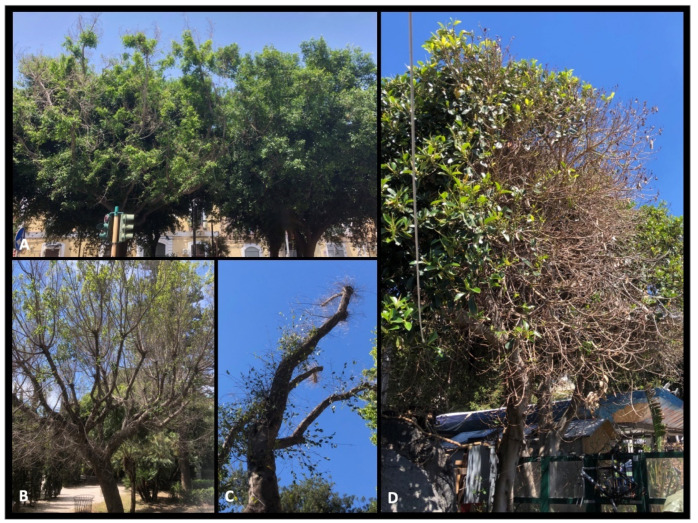
Symptoms of *Botryosphaeriaceae* disease observed in urban areas on *F. microcarpa*. (**A**) Diseased (left) and healthy (right) plants. (**B**–**D**) Defoliation and shoot dieback all over the canopy. (**C**) New twigs growing below the dead shoots.

**Figure 2:**
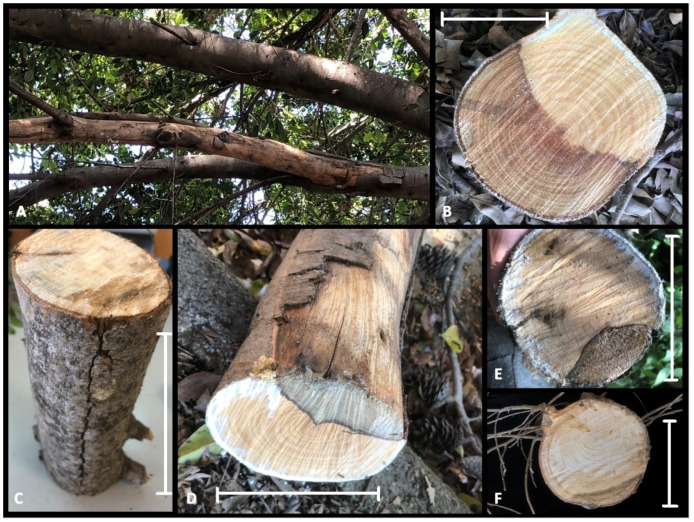
Internal symptoms. (**A**) Dead branch showing cracking of the outer layers of the bark (upper), healthy branch (lower). (**B**–**F**) Internal cankers and bark cracked along the branch with diseased tissue sharply demarcated from adjacent, healthy wood. Scale bars: (**B**) = 15 cm; (**C**) = 50 cm; (**D**–**F**) = 20 cm.

*Ficus microcarpa* growing in a wide range of site conditions (tree-lined streets, gardens, public parks, and squares) have suffered a widespread dieback in Catania and Siracusa provinces. In the public areas where the research was conducted, more than 40 mature *F. microcarpa* trees (20 to 50 years old) showed cankered twigs and branches on approximately 450 plants. The trees still appeared green in part of the canopy, although this was accompanied by parts of branches and shoots that were defoliated and dead (Figure 1A–D and Figure 2A). Sometimes, it was possible to observe new twigs growing below the damaged branches (Figure 1C). The sample consisted of large portions of branches showing severe internal wood discolouration, including of sapwood and the heartwood (Figure 2B–F). Often, the bark appeared cracked and split along the branches (Figure 2), and internal cankers were sharply demarcated from adjacent, healthy wood (Figure 2B–F). Isolations frequently (>70%) yielded *Botryosphaeriaceae*-like fungi, characterized, as reported by Slippers and Wingfield [11], by a ‘fluffy’ mycelium, either white-to-creamy, pigmented ‘greenish brown’, or gray-to-gray-black. Moreover, with lower frequencies, colonies of *Eutypella*-like species were also isolated from symptomatic tissues.

### 2.2. Morphological Characterization and Phylogenetic Analysis

**Figure 3:**
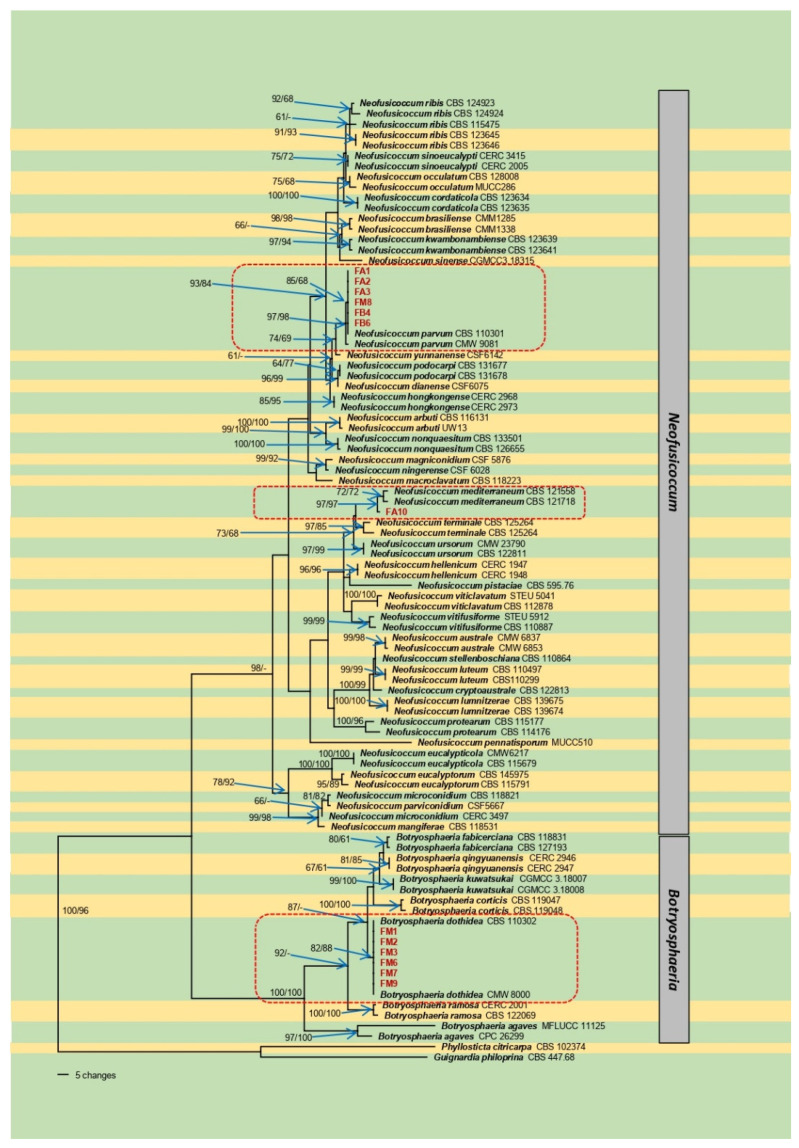
One of 100 equally parsimonious trees generated from maximum-parsimony analysis of the three-gene (ITS + *tef1*-α + *tub2*) combined dataset from *Botryosphaeriaceae* species. Numbers in front and after the slash represent parsimony and likelihood bootstrap values from 1000 replicates, respectively. Isolates in red were generated in this study. Bar indicates the number of nucleotide changes.

The PCR amplification of the ITS region, *tef1*-α, and *tub2* generated 577 to 581, 273 to 288, and 422 to 446 bp fragments, respectively. The phylogenetic analyses were performed using a dataset of the three concatenated loci. The sequences generated in this study were deposited in GenBank (Table 1). A preliminary comparison of our sequences in GenBank showed our isolates belonging to the genera *Botryosphaeria* and *Neofusicoccum*. The *Eutypella*-like species showed high similarity with different *Eutypella* species submitted to GenBank. Since these isolates were excluded from the phylogenetic analyses due to their results in the pathogenicity test, they were identified as *Eutypella* spp. The phylogenetic analyses were then conducted only for the *Botryosphaeriaceae*. The results of the partition-homogeneity test indicated no (*p* = 1.00) significant differences in the three-gene dataset. The MP analysis of the combined dataset showed that of 2921 total characters, 391 were parsimony-informative, 220 were parsimony-uninformative, and 2310 were constant. In total, 100 trees were retained. Tree length was equal to 1098, CI = 0.707, RI = 0.912, RC = 0.644. The best-fit model of nucleotide evolution based on the AIC was GTR + I + G for ITS, GTR + G for *tef1*-α, and HKY + G for *tub2*. The ML analysis showed that of 2921 total characters, 2310 were constant, 475 were parsimony informative, and 136 were autapomorphic. The results of both analyses showed that the isolates FM1-3, FM6 and 7, and FM9 were grouped in the clade of *B. dothidea* (82/88, MP and ML bootstrap support %, respectively), the isolate FA10 grouped within *N. mediterraneum* clade (97/97), and FA1-3, FM8, FB4, and FB6 were grouped with the clade of *N. parvum* (97/98) (Figure 3). The conidia measurements were (18.66)–22.7–(28.34) × (3.61)–4.9–(6.38) for *B. dothidea,* (14.0)–20.0–(27.2) × (4.3)–5.8–(6.8) for *N. mediterraneum*, and (12.78)–15.1–(16.9) × (4.16)–5.3–(7.21) for *N. parvum.*

### 2.3. Pathogenicity Test

**Figure 4:**
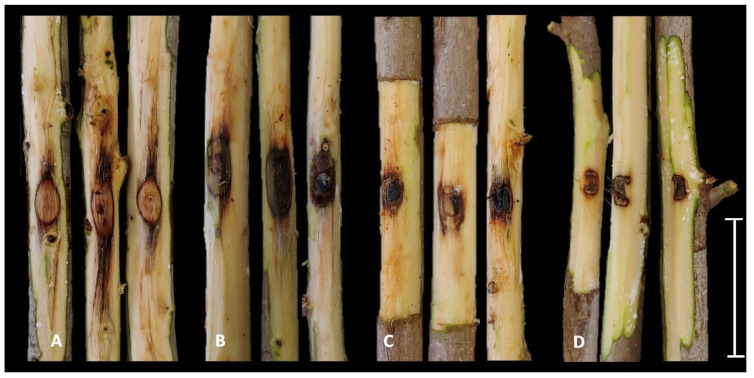
Results of pathogenicity test after two weeks. (**A**) *Neofusicoccum mediterraneum*. (**B**) *N. parvum*. (**C**) *Botryosphaeria dothidea*. (**D**) Control. Scale bar = 10 cm.

**Figure 5:**
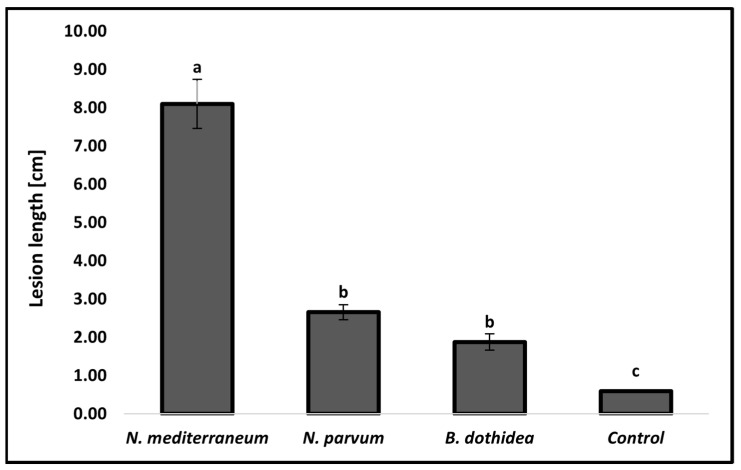
Comparisons of average lesion length (cm) resulting from pathogenicity test among *B. dothidea*, *N. mediterraneum* and *N. parvum* on potted plants. Columns are the means of 15 inoculation points (five per plant) for each fungal species. Control consisted of 12 inoculation points. Vertical bars represent the standard error of the means. Bars topped with different letters indicate treatments that were significantly different according to Fisher’s protected LSD test (α = 0.05).

The results of the pathogenicity test showed that all three species of *Botryosphaeriaceae* identified in this study were pathogenic to *F. microcarpa*. Otherwise, the *Eutypella* sp. isolate inoculated did not induce any lesions on the woody tissues, which was similar to the control. For this reason, this species was excluded from the phylogenetic analyses. External discoloration out of the inoculation point was observed after 7 days and all the inoculated trees showed severe wood discoloration after the outer layer of bark was removed (Figure 4A–D) Moreover, young twigs close to the inoculation point rapidly wilted a few days after inoculation. Specifically, among the fungal species, the *N. mediterraneum* isolate FA10 induced the longest lesions (mean 8.10 cm), followed by *N. parvum* isolate FB4 (2.66 cm) and *B. dothidea* isolate FM2 (1.88 cm). All the inoculated species statistically differed from the control (*p* < 0.05) (Figure 5). The colonies that emerged from the re-isolations showed morphological characteristics (color, shape, and mycelium texture) that fulfilled the Koch’s postulates.

## 3. Discussion

The results of our study confirm, for the first time, the presence of three species, *B. dothidea*, *N. mediterraneum*, and *N. parvum*, affecting *F. microcarpa* in Italy. Regarding *Botryosphaeriaceae*, little is known about its association with *F. microcarpa*. According to the U.S. National Fungus Collections Fungal Database [9], only a few, old reports describe the association of *Lasiodiplodia theobromae* (as *Botryodiplodia theobromae*) in Pakistan [31] and Egypt [29], and *Diplodia fici-retusae* in Taiwan on *Ficus retusa* (synonymous of *F. microcarpa*) [32,33]. A commonly reported disease of *F. microcarpa*, as well as other *Ficus* spp., is “sooty canker”, which is caused by *Neoscytalidium dimidiatum* (traditionally reported also as *Hendersonula toruloidea* and *Natrassia mangiferae*). The pathogen, as well as other *Botryosphaeriaceae*, induces cankers and dieback, often accompanied by a powdery mass of black spores (arthroconidia) produced by this species [14,18,19,20,21,26,28,34]. Recently, in California *B. dothidea*, *N. luteum*, *N. mediterraneum*, and *N. parvum* were reported as causing branch cankers and dieback on *F. microcarpa* trees in Los Angeles County [25]. In recent years, *Botryosphaeriaceae* spp. have been reported attacking many different crops in Italy, and, especially in Sicily, it is well known that these species spread from nurseries to the open field, from ornamental plants to the agricultural ones. Specifically, *B. dothidea* has recently been reported in Sicily on walnut and pistachio [35,36]. Moreover, *N. mediterraneum* and *N. parvum* have been reported as highly aggressive pathogens among the *Botryosphaeriaceae* in Sicily [13,35,37,38]. In addition, *N. mediterraneum* was the most encountered species in Sicilian pistachio orchards [36]. From this and previous studies conducted in Sicily, it emerged that *Botryosphaeriaceae* spp., and especially the species described in this study, are easily encountered in different hosts and landscapes. Regarding the ecology of these fungi, it is well known that they are also endophytes on many hosts [11], often coexisting in the same tissues [39] and forming long latent infections [40,41]. This must be taken into serious consideration, since many infections can spread from nurseries (as latent infections) to open fields. Recently, studies conducted in California on latent infections on nut crops helped us to properly quantify these pathogens using real-time PCR assays [40,42,43]. The ability of these fungi to disperse their spores (conidia) by wind, rain, and insects [10] in conjunction with intercontinental human movements with no adequate quarantine strategies led them to easily spread all over the world [44], as demonstrated for *N. parvum*, the most adapted organism, which is detected from the north to the south, excluding boreal forests and montane grasslands [45]. Many factors can be involved in the ability of some *Botryosphaeriaceae* species to jump from one host to another, meaning that they are more virulent than other species. Among these, a recent study [46] revealed how some groups of taxa, such as *Botryosphaeria*, *Lasiodiplodia*, and *Neofusicoccum*, show an expansion of certain clades of gene families involved in the pathogenesis. Specifically, in the *Botryosphaeria* and *Neofusicoccum* genomes, an expansion of secreted cell-wall-degrading enzymes (CAZymes) was observed [46]. It is no surprise that the species identified in this study also occurred on other taxonomically distant hosts in Sicily. Batista et al. [45] showed that *B. dothidea* is associated with 403 hosts in 66 countries, and *N. parvum* with 223 hosts in 50 countries. In recent decades, in Sicily, a relevant increase was observed in *Botryosphaeriaceae* in nurseries, as well as in open fields (Polizzi G., unpublished data). *Botryosphaeriaceae* disease expression is strongly related to stresses due to factors other than the *Botryosphaeriaceae* infection itself [47,48,49]. Related to this, it should be noticed that climate change contributes to additional stress or pressure on woody plants through extreme weather conditions or the expansion of pathogens’ host ranges [11]. In fact, climate change affects the dynamics of fungal populations, in terms of biology and ecology [49]. Gange et al. [50] conducted a study in the UK on the species *Auricularia auricula-judae*, demonstrating an alteration in the phenology (the earlier appearance of fruit bodies and a longer fruiting period) and an expansion of the host range consistent with a response to observed warming trends in the climate, also suggesting that climate change affected the interactions between wood-inhabiting fungi. Combative interactions are considered the main drivers of fungal community development in decaying wood [51,52], and these can be strongly affected by temperature, water potential, gaseous regime, and resource size [53,54,55]. All these factors contribute to making *Botryosphaeriaceae* disease severe and ubiquitous, compared with otherwise “mild diseases” [56]. Urban areas, which are even less investigated than agricultural ones, must be considered crucial routes of introduction and dissemination for *Botryosphaeriaceae* [57]. It is well known that stressed trees are much more predisposed to *Botryosphaeriaceae* disease [11,58], and this should be taken into careful consideration regarding ornamental trees in urban landscapes. In fact, trees grown in urban areas can also be considered more exposed to stress factors [59], and thus more susceptible to *Botryosphaeriaceae* disease. This could represent a serious threat in urban areas, not only in terms of aesthetic damage, but mostly in terms of public safety. In relation to these predisposing factors, we ascertained during our investigation that *F. microcarpa* trees grown in the urban areas of Catania and Siracusa provinces were severely and improperly pruned, especially during the humid seasons. In order to avoid the spread of *Botryosphaeriaceae* species, some recommendations should be taken into serious consideration. Since it is known that both rainfall and fog [60,61] positively affect the release of *Botryosphaeriaceae* spores, farmers or pruning crews should not prune when rain is forecasted or with dense fog to avoid the contamination of fresh wounds by *Botryosphaeriaceae* [62]. Moreover, recommendations as to pruning type depend on the tree species, which is why trained pruning crews should be selected for this crucial practice. As demonstrated on pistachio, Botryosphaeria panicle and shoot blight were reduced by 50–60% by trained pruning crews compared to the disease levels in trees pruned by unspecialized crews [63]. Furthermore, in California, field experiments conducted on *F. carica* affected by fig limb dieback demonstrated that pruning 5 cm below the canker successfully removed the pathogen from the tissues [36]. Regarding trained pruning crews, it is crucial that workers disinfect their pruning tools, since these could easily transmit inoculum (spores, mycelium, and fruit bodies) from one tree to another. As demonstrated on walnut, pathogen spores were transferred from the chainsaws to the agar media, whereas *Botryosphaeriaceae* species were not found when the chainsaws were disinfected with a 2% dilution of vinegar or commercial household bleach (T.J. Michailides, unpublished data/personal communication). In addition, the usage of biological control agents as protectants for pruning wounds, especially in urban areas, should be considered. Encouraging results have been obtained on other crops, such as almond and grapevine treated with *Trichoderma*-based formulants against canker pathogens [64,65,66]. Further investigations need to be conducted in this direction. Good agronomic practices and, possibly, the usage of biocontrol agents, can help us to control *Botryosphaeriaceae* disease in urban areas. To our knowledge, this is the first study of *Botryosphaeriaceae* disease on *F. microcarpa* in Europe.

## 4. Materials and Methods

### 4.1. Surveys and Fungal Isolations

During the years between 2019 and 2020, surveys were carried out in numerous urban areas of the cities of Catania (Catania province), and Siracusa (Siracusa province), Sicily, where *F. microcarpa* were the most prevalent ornamental trees, including tree-lined streets, gardens, public parks, and squares. Several symptomatic samples obtained from ten plants were collected and brought to the laboratory of the Dipartimento di Agricoltura, Alimentazione e Ambiente, University of Catania, for further investigations. For culture isolation, small sections (0.2 to 0.3 cm^2^) of symptomatic tissues (branches and shoots) were surface-disinfected for 1 min in 1.5% sodium hypochlorite, rinsed in sterile water, dried on sterile absorbent paper under laminar hood and placed on potato dextrose agar (PDA, Lickson, Vicari, Italy) amended with 100 mg/liter of streptomycin sulfate (Sigma-Aldrich, St. Louis, MO, USA) (PDAS) to prevent bacterial growth, and then incubated at 25 ± 1 °C for 3–5 days until fungal colonies were large enough to be examined. Subsequently, colonies of interest were transferred to fresh PDAS to make pure cultures, and then single-hyphal tip cultures were obtained and maintained on PDAS at 25 ± 1 °C. Isolates characterized in this study were stored in the fungal collection of the laboratory with the labels FA, FB, and FM.

### 4.2. Morphological and Molecular Characterization

For the morphological characterization of the pathogens, the length and width of 50 conidia from the 21-day-old colonies of the isolates FM1, FA10, and FA1 grown on PDA were measured using a fluorescence microscope (Olympus-BX61) coupled to an Olympus DP70 digital camera; measurements were captured using software analysis 3.2 (Soft Imaging System GmbH, Münster, Germany). Dimensions are reported as the minimum and maximum in parentheses and the average. Total fungal DNA was extracted using the Wizard Genomic DNA Purification Kit (Promega Corporation, Madison, WI, USA), scraping the mycelium with a sterile scalpel from 5-day-old fungal cultures grown on PDA or malt extract agar (MEA, Oxoid LTD. Basingstoke, Hampshire, England) media. The genomic DNA extracted was visualized on 1% agarose gels (90 V for 40 min) stained with GelRed^®^ (Biotium, Fremont, CA, USA). The quality of the DNA was determined through Nanodrop Lite Spectrophotometer (Thermo Fisher Scientific, Wilmington, DE, USA). The internal transcriber spacer region (ITS) of the nuclear ribosomal RNA operon was amplified with primers ITS5 (5′-GGA AGT AAA AGT CGT AAC AAG G-3′) and ITS4 (5′-TCC TCC GCT TAT TGA TAT GC-3′) [67]; the primers EF1-728F (5′-CAT CGA GAA GTT CGA GAA GG-3′) and EF1-986R (5′-TAC TTG AAG GAA CCC TTA CC-3′) [68] were used to amplify part of the translation elongation factor 1alpha gene (*tef1*-α); and primer sets Bt2a (5′-GGT AAC CAA ATC GGT GCT TTC-3′) and Bt2b (5′-ACC CTC AGT GTA GTG ACC CTT GGC-3′) [69] were used for the partial beta tubulin (*tub2*). Amplification by polymerase chain reaction (PCR) was performed in a total volume of 25 µL using One Taq^®^ 2X Master Mix with Standard Buffer (BioLabs, New England, NEB), according to the manufacturer’s instructions, on an Eppendorf Mastercycler (AG 22331 Hamburg, Germany). The thermal cycle consisted of initial 30 s at 94 °C, followed by 35 cycles at 94 °C for 30 s, 49 °C (ITS), 57–59 °C (*tef1*-α), or 52 °C (*tub2*) for 1 min, 68 °C for 1 min, and 5 min at 68 °C. Regarding *Botryosphaeriaceae*, in total, 45 isolates were sequenced (*tub2*) and only 13 representative isolates were considered for further gene sequencing and phylogenetic analyses. Concerning the *Eutypella*-like species, a total of 7 isolates were sequenced (ITS and *tub2*). PCR products were visualized on 1% agarose gels (90 V for 40 min), purified, and sequenced by Macrogen Inc. (Seoul, South Korea). Forward and reverse DNA sequences were assembled and edited using MEGA X: Molecular Evolutionary Genetics Analysis across computing platforms [70] and submitted to GenBank.

### 4.3. Phylogenetic Analysis

Chromatograms were viewed using FinchTV Version 1.4.0 (Geospiza, Inc.; Seattle, WA, USA; http://www.geospiza.com (accessed on 16 February 2022)). Sequences were read and edited using MEGAX. Before constructing the phylogenetic tree, BLAST searches were performed using the NCBI nucleotide database [71]. ITS, *tef1*-α, and *tub2* DNA sequence datasets were aligned using MEGAX, and manual alignments were performed when necessary. A partition-homogeneity test with heuristic search and 1000 homogeneity replicates was performed using PAUP* (Phylogenetic Analysis Using Parsimony) version 4.0a (Sinauer Associates, Sunderland, MA, USA) [72] to test for discrepancies in the three-gene dataset. For comparison, 79 additional sequences were selected according to the recent literature on the *Botryosphaeriaceae* [73,74] to be included in the alignment (Table 1). Maximum parsimony analysis (MP) was performed in PAUP v.4.0a. The analysis of the combined dataset (ITS + *tef1*-α + *tub2*) was performed with the heuristic search function and tree bisection and reconstruction (TBR) as branch-swapping algorithms with the branch-swapping option set to ‘best trees’ only. Gaps were treated as ‘missing’, the characters were unordered and of equal weight, and Maxtrees were limited to 100. Tree length (TL), consistency index (CI), retention index (RI), and rescaled consistency index (RC) were calculated. To identify the best-fit model of nucleotide evolution for each gene according to the Akaike information criterion (AIC), MrModeltest v. 2.4 [75] was used. The maximum likelihood analysis (ML) of the combined genes was performed in GARLI v.0.951 [76]. For both analyses, clade support was assessed by 1000 bootstrap replicates. *Guignardia philoprina* (CBS 447.68) and *Phyllosticta citricarpa* (CBS 102374) served as the outgroup in both analyses.

### 4.4. Pathogenicity Test

Pathogenicity tests were conducted on potted, healthy, 4-year-old *F. microcarpa* plants maintained at room temperature. For each fungal species, one representative isolate was inoculated. Specifically, three plants were used for each isolate, and five inoculation points were chosen along the trunk on each plant (~30 cm distant one from each other). The inoculation site was first surface-disinfected by spraying with 70% ethanol solution, and wounds were made with a sterilized 6-millimeter cork borer after removing the bark, and a mycelium plug (6 mm in diameter) was placed upside down into the plant tissue wound. Wounds were sealed with Parafilm^®^ (Pechney Plastic Packaging Inc., Chicago, IL, USA). In total, 12 additional wounds were inoculated with sterile PDA plugs as controls. Plants were regularly watered. The presence and length of the resulting lesions were recorded two weeks after the inoculation. Lesion length measurements were analyzed in Statistix 10 [77] via analysis of variance (ANOVA), and mean differences were compared with the Fisher’s protected least significant difference (LSD) test at α = 0.05. In order to fulfil Koch’s postulates, re-isolations were carried out on PDAS following the procedure described above. Each re-isolated fungus was identified through the observation of colony characteristics.

## 5. Conclusions

In the present study, three species of *Botryosphaeriaceae* were isolated from symptomatic samples of *F. microcarpa* showing severe symptoms of cankers, wood discolourations, bark cracking, and dieback. Morphological and molecular tools identified *B. dothidea*, *N. mediterraneum*, and *N. parvum*. Pathogenicity tests fulfilled Koch’s postulates. The results of this study provide new information on this important family of phytopathogenic fungi and its wide host range. This is the first report in Europe of *Botryosphaeriaceae* affecting *F. microcarpa*.

## Figures and Tables

**Table 1:** Information on fungal isolates used in the phylogenetic analyses and their corresponding GenBank accession numbers. Isolates in bold are from this study.

Scheme 1.	Isolate ID	ITS	*tef1*-α	*tub2*
*Botryosphaeria agaves*	CBS 133992 = MFLUCC 11-0125T	JX646791	JX646856	JX646841
*B. agaves*	CBS 141505 = CPC 26299	KX306750	MT592030	MT592463
*B. corticis*	CBS 119047T	DQ299245	EU017539	EU673107
*B. corticis*	CBS 119048 = CAP 198	DQ299246	EU017540	MT592464
*B. dothidea*	CBS 115476 = CMW 8000T	AY236949	AY236898	AY236927
*B. dothidea*	CBS 110302 = CAP 007	AY259092	AY573218	EU673106
*B. dothidea*	**FM1**	**OM241975**	**OM262426**	**OM262439**
*B. dothidea*	**FM2**	**OM241976**	**OM262427**	**OM262440**
*B. dothidea*	**FM3**	**OM241977**	**OM262428**	**OM262441**
*B. dothidea*	**FM6**	**OM241978**	**OM262429**	**OM262442**
*B. dothidea*	**FM7**	**OM241979**	**OM262430**	**OM262443**
*B. dothidea*	**FM9**	**OM241980**	**OM262431**	**OM262444**
*B. fabicerciana*	CBS 118831 = CMW 14009	DQ316084	MT592032	MT592468
*B. fabicerciana*	CBS 127193 = CMW 27094T	HQ332197	HQ332213	KF779068
*B. kuwatsukai*	CGMCC 3.18007	KX197074	KX197094	KX197101
*B. kuwatsukai*	CGMCC 3.18008	KX197075	KX197095	KX197102
*B. qingyuanensis*	CERC 2946 = CGMCC 3.18742T	KX278000	KX278105	KX278209
*B. qingyuanensis*	CERC 2947 = CGMCC 3.18743	KX278001	KX278106	KX278210
*B. ramosa*	CERC 2001 = CGMCC 3.187396	KX277989	KX278094	KX278198
*B. ramosa*	CBS 122069 = CMW 26167T	EU144055	EU144070	KF766132
*Guignardia philoprina*	CBS 447.68	FJ824768	FJ824773	FJ824779
*Neofusicoccum arbuti*	CBS 116131 = AR 4014T	AY819720	KF531792	KF531793
*N. arbuti*	CBS 117090 = UW13	AY819724	KF531791	KF531794
*N. australe*	CBS 139662 = CMW 6837T	AY339262	AY339270	AY339254
*N. australe*	CMW 6853	AY339263	AY339271	AY339255
*N. brasiliense*	CMM 1285	JX513628	JX513608	KC794030
*N. brasiliense*	CMM 1338T	JX513630	JX513610	KC794031
*N. cordaticola*	CBS 123634 = CMW 13992T	EU821898	EU821868	EU821838
*N. cordaticola*	CBS 123635	EU821903	EU821873	EU821843
*N. cryptoaustrale*	CBS 122813 = CMW 23785T	FJ752742	FJ752713	FJ752756
*N. dianense*	CSF6075 = CGMCC3.20082T	MT028605	MT028771	MT028937
*N. eucalypticola*	CBS 115679 = CMW 6539T	AY615141	AY615133	AY615125
*N. eucalypticola*	CBS 115766 = CMW 6217	AY615143	AY615135	AY615127
*N. eucalyptorum*	CBS 115791 = CMW 10125 = BOT 24T	AF283686	AY236891	AY236920
*N. eucalyptorum*	CBS 145975 = CPC 29337	MT587477	MT592190	MT592682
*N. hellenicum*	CERC 1947 = CFCC 50067T	KP217053	KP217061	KP217069
*N. hellenicum*	CERC 1948 = CFCC 50068	KP217054	KP217062	KP217070
*N. hongkongense*	CERC2973 = CGMCC3.18749T	KX278052	KX278157	KX278261
*N. hongkongense*	CERC 2968 = CGMCC 3.18748	KX278051	KX278156	KX278260
*N. kwambonambiense*	CBS 123639 = CMW 14023T	EU821900	EU821870	EU821840
*N. kwambonambiense*	CBS 123641 = CMW 14140	EU821919	EU821889	EU821859
*N. lumnitzerae*	CBS 139674 = CMW 41469T	KP860881	KP860724	KP860801
*N. lumnitzerae*	CBS 139675 = CMW 41228	MT587480	MT592193	MT592685
*N. luteum*	CBS 110497 = CPC 4594 = CAP 037	EU673311	EU673277	EU673092
*N. luteum*	CBS 110299 = LM 926 = CAP 002T	AY259091	KX464688	DQ458848
*N. macroclavatum*	CBS 118223 = CMW 15955 = WAC 12444T	DQ093196	DQ093217	DQ093206
*N. magniconidium*	CSF5876 = CGMCC3.20077T	MT028612	MT028778	MT028944
*N. mangiferae*	CBS 118531 = CMW 7024T	AY615185	DQ093221	AY615173
*N. mediterraneum*	CBS 121558	GU799463	GU799462	GU799461
*N. mediterraneum*	CBS 121718 = CPC 13137T	GU251176	GU251308	GU251836
*N. mediterraneum*	**FA10**	**OM241968**	**OM241976**	**OM262432**
*N. microconidium*	CERC3497 = CGMCC3.18750T	KX278053	KX278158	KX278262
*N. microconidium*	CBS 118821 = CMW 13998	MT587497	MT592212	MT592704
*N. ningerense*	CSF6028 = CGMCC3.20078T	MT028613	MT028779	MT028945
*N. nonquaesitum*	CBS 126655 = L3IE1 = PD484T	GU251163	GU251295	GU251823
*N. nonquaesitum*	CBS 133501 = UCR532	MT587498	MT592213	MT592705
*N. occulatum*	CBS 128008 = MUCC 227T	EU301030	EU339509	EU339472
*N. occulatum*	MUCC 286 = WAC 12395	EU736947	EU339511	EU339474
*N. parvum*	CBS 138823 = ICMP 8003 = CMW 9081T	AY236943	AY236888	AY236917
*N. parvum*	CBS 110301 = CAP 074	AY259098	AY573221	EU673095
*N. parvum*	**FA1**	**OM241969**	**OM262420**	**OM262433**
*N. parvum*	**FA2**	**OM241970**	**OM262421**	**OM262434**
*N. parvum*	**FA3**	**OM241971**	**OM262422**	**OM262435**
*N. parvum*	**FM8**	**OM241972**	**OM262423**	**OM262436**
*N. parvum*	**FB4**	**OM241973**	**OM262424**	**OM262437**
*N. parvum*	**FB6**	**OM241974**	**OM262425**	**OM262438**
*N. parviconidium*	CSF5667 = CGMCC3.20074T	MT028615	MT028781	MT028947
*N. pennatisporum*	WAC 13153 = MUCC 510T	EF591925	EF591976	EF591959
*N. pistaciae*	CBS 595.76T	KX464163	KX464676	KX464953
*N. podocarpi*	CBS 131677 = CMW 35494	MT587508	MT592223	MT592715
*N. podocarpi*	CBS 131678 = CMW 35499	MT587509	MT592224	MT592716
*N. protearum*	CBS 114176 = CPC 1775 = JT 189T	AF452539	KX464720	KX465006
*N. protearum*	CBS 115177 = CPC 4357	FJ150703	MT592239	MT592731
*N. ribis*	CBS 115475 = CMW 7772T	AY236935	AY236877	AY236906
*N. ribis*	CBS 124923 = CMW 28320	FJ900608	FJ900654	FJ900635
*N. ribis*	CBS 124924T	FJ900607	FJ900653	FJ900634
*N. ribis*	CBS 123645 = CMW 14058T	EU821904	EU821874	EU821844
*N. ribis*	CBS 123646 = CMW 14060	EU821905	EU821875	EU821845
*N. sinense*	CGMCC3.18315T	KY350148	KY817755	KY350154
*N. sinoeucalypti*	CERC2005 = CGMCC3.18752T	KX278061	KX278166	KX278270
*N. sinoeucalypti*	CERC3415	KX278063	KX278168	KX278272
*N. stellenboschiana*	CBS 110864 = CPC 4598	AY343407	AY343348	KX465047
*N. terminaliae*	CBS 125263 = CMW 26679T	GQ471802	GQ471780	KX465052
*N. terminaliae*	CBS 125264 = CMW 26683	GQ471804	GQ471782	KX465053
*N. ursorum*	CBS 122811 = CMW 24480T	FJ752746	FJ752709	KX465056
*N. ursorum*	CBS 122812 = CMW 23790	FJ752745	FJ752708	KX465057
*N. yunnanense*	CSF6142 = CGMCC3.20083T	MT028667	MT028833	MT028999
*N. viticlavatum*	CBS 112878 = CPC 5044 = JM 86T	AY343381	AY343342	KX465058
*N. viticlavatum*	CBS 112977 = STE-U 5041	AY343380	AY343341	KX465059
*N. vitifusiforme*	CBS 110887 = CPC 5252 = JM5T	AY343383	AY343343	KX465061
*N. vitifusiforme*	CBS 121112 = STE-U 5912	EF445349	EF445391	KX465016
*Phyllosticta citricarpa*	CBS 102374	FJ824767	FJ538371	FJ824778

T: Type material.

## Data Availability

The data presented in this study are available on request from the corresponding author.
